# Racial Disparities in Obesity‐Related Cardiovascular Mortality in the United States: Temporal Trends From 1999 to 2020

**DOI:** 10.1161/JAHA.122.028409

**Published:** 2023-09-06

**Authors:** Zahra Raisi‐Estabragh, Ofer Kobo, Jennifer H. Mieres, Renee P. Bullock‐Palmer, Harriette G.C. Van Spall, Khadijah Breathett, Mamas A. Mamas

**Affiliations:** ^1^ William Harvey Research Institute, NIHR Barts Biomedical Research Centre, Centre for Advanced Cardiovascular Imaging Queen Mary University London United Kingdom; ^2^ Barts Heart Centre, St. Bartholomew’s Hospital Barts Health NHS Trust, West Smithfield London United Kingdom; ^3^ Keele Cardiovascular Research Group Keele University Keele United Kingdom; ^4^ Department of Cardiology Hillel Yaffe Medical Center Hadera Israel; ^5^ Department of Cardiology, Hofstra Northwell School of Medicine Hofstra University, Lake Success New York NY USA; ^6^ Department of Cardiology Deborah Heart and Lung Center Brown Mills NJ USA; ^7^ Department of Medicine, Department of Health Research Methods, Evidence, and Impact, Population Health Research Institute Research Institute of St. Joe’s, McMaster University Hamilton Ontario Canada; ^8^ Division of Cardiovascular Medicine, Department of Medicine Indiana University Indianapolis IN USA; ^9^ Institute of Population Health University of Manchester Manchester United Kingdom

**Keywords:** body mass index, cardiovascular disease, epidemiology, ethnicity, health inequalities, public health, Epidemiology, Cardiovascular Disease, Disparities, Obesity

## Abstract

**Background:**

Obesity is a major risk factor for cardiovascular disease, with differential impact across populations. This descriptive epidemiologic study outlines trends and disparities in obesity‐related cardiovascular mortality in the US population between 1999 and 2020.

**Methods and Results:**

The Multiple Cause of Death database was used to identify adults with primary cardiovascular death and obesity recorded as a contributing cause of death. Cardiovascular deaths were grouped into ischemic heart disease, heart failure, hypertensive disease, cerebrovascular disease, and other. Absolute, crude, and age‐adjusted mortality rates (AAMRs) were calculated by racial group, considering temporal trends and variation by sex, age, and residence (urban versus rural). Analysis of 281 135 obesity‐related cardiovascular deaths demonstrated a 3‐fold increase in AAMRs from 1999 to 2020 (2.2‐6.6 per 100 000 population). Black individuals had the highest AAMRs. American Indian or Alaska Native individuals had the greatest temporal increase in AAMRs (+415%). Ischemic heart disease was the most common primary cause of death. The second most common cause of death was hypertensive disease, which was most common in the Black racial group (31%). Among Black individuals, women had higher AAMRs than men; across all other racial groups, men had a greater proportion of obesity‐related cardiovascular mortality cases and higher AAMRs. Black individuals had greater AAMRs in urban compared with rural settings; the reverse was observed for all other races.

**Conclusions:**

Obesity‐related cardiovascular mortality is increasing with differential trends by race, sex, and place of residence.

**Nonstandard Abbreviations and Acronyms**
CDC WONDER: Centers for Disease Control and Prevention Wide‐Ranging Online Data for Epidemiologic Research

**Clinical Perspective**

**What Is New?**

- In this analysis of population‐level US mortality data, we observed a 3‐fold increase in obesity‐related cardiovascular age‐adjusted mortality rates between 1999 and 2020.
- The most common primary cardiovascular causes of death related to obesity were ischemic heart disease and hypertensive diseases. Black individuals had higher obesity‐related cardiovascular age‐adjusted mortality rates than any other racial group throughout the study period.
- Across all racial groups, obesity‐related cardiovascular age‐adjusted mortality rates were greater in rural compared with urban settings, except in Black individuals, who had higher age‐adjusted mortality rates in urban settings.

**What Are the Clinical Implications?**

- The impact of obesity on cardiovascular health is increasing, with some groups affected more than others.
- The observed population disparities provide insights for implementation of effective prevention strategies both at the population and individual level.
- Such interventions should incorporate strategies to address sociopolitical sources of health inequalities for individual communities with the aim of alleviating the burden of both obesity and cardiovascular disease.

The rising prevalence of obesity is a global public health crisis. In the United States between 2017 and 2020, the estimated prevalence of obesity was 41.9%, an increase of almost 10% from the preceding decade.^1^ Similar trends are reported in almost every country worldwide.^2^ Obesity is linked to increased risk of a multitude of noncommunicable diseases,^3^ with the medical cost of obesity in the United States estimated at $173 billion.^4^

Excess weight gain is driven by a complex interconnected network of genetic, physiologic, and environmental exposures.^5^ Thus far, obesity prevention and treatment interventions, both at the individual and population level, have had minimal long‐term success.^3^ Many of the drivers of obesity depend on where and how people live, which determines access to education, health care, healthy food, and safe places to be active.^6^, ^7^ Obesity affects some people more than others and is strongly associated with socioeconomic disenfranchisement, which is more commonly encountered in underrepresented racial and ethnic groups. Structural racism impacts on experiences throughout the life course and can limit access to housing, education, employment, and health care.

Obesity is a major risk factor for cardiovascular disease (CVD),^8^, ^9^ the most common cause of ill health and death in the world.^10^ Although several reports have highlighted racial differences in the prevalence of obesity,^1^, ^11^ the links between such variations and cardiovascular mortality has not been previously reported. Dedicated study of such relationships and trends is paramount to understanding societal causes of obesity and to guide strategies that may alleviate the population burden of both obesity and CVD.

The aim of the present study was to describe racial differences in obesity‐related cardiovascular mortality in the US population between 1999 and 2020, considering temporal trends and variation by sex, age, and place of residence (rural versus urban).

## Methods

The data underlying this article are available through the CDC WONDER (Centers for Disease Control and Prevention Wide‐Ranging Online Data for Epidemiologic Research) resource at: https://wonder.cdc.gov/mcd‐icd10.html.

### Ethics and Consent

The analysis uses anonymized data. Ethical approval and consent were not required.

### Setting and Study Population

The Multiple Cause of Death database includes mortality and population counts from all US counties between 1999 and 2020. The cause of death extracted from physician‐completed death certificates is available for all US residents. This includes a single underlying cause of death and up to 20 contributing causes of death recorded using *International Classification of Diseases, Tenth Revision* (*ICD‐10*) codes. The cause of death information is linked to key demographic data. The number of deaths, crude death rates, age‐adjusted death rates, and 95% CI for death rates can be obtained by cause of death, place of residence, age, sex, race, and year.

### Analysis Sample

We included deaths in adults (>15 years old) occurring between 1999 and 2020. We selected individuals with CVD (*ICD‐10* codes: I00–I99) recorded as the primary (underlying) cause of death and obesity (*ICD‐10* code: E66) recorded as a contributing cause of death. We categorized CVD into ischemic heart disease (*ICD‐10* codes: I20–I25), heart failure/cardiomyopathy (*ICD‐10* codes: I42, I50), hypertensive diseases (*ICD‐10* codes: I10–I15), cerebrovascular disease (*ICD‐10* codes: I60–I69), and other CVDs (composite of all CVDs not captured in other defined categories).

### Characterization of Racial Groups

The CDC WONDER resource provides bridged‐race population data within 4 racial categories: Asian or Pacific Islander, Black, American Indian or Alaska Native, and White. This information is updated annually from the National Center for Health Statistics bridged‐race population estimates.^12^ The estimates result from bridging the 31 race categories used in Census 2000, as indicated in the Office of Management and Budget standards for the collection of data on race and ethnicity, to the 4 specific racial categories specified under the 1997 Office of Management and Budget standards for the collection of data on race and ethnicity.

### Statistical Analysis

We calculated obesity‐related overall and disease‐specific cardiovascular mortality measures (absolute, crude, and age‐adjusted rates) for each racial group. Results were stratified by sex (men, women), age (10‐year age groups), urbanization status (urban‐large, medium, and small metropolitan areas versus rural‐nonmetropolitan area counties according to the 2013 US Census classification), and region of residence (Northeast, Midwest, South, and West). Age‐adjusted mortality rates are presented per 100 000 population. Mortality rates were calculated for each year. The population estimates used as the denominators of rates were specific to race, sex, age groups, and place of death. The AAMRs for each year were obtained from the CDC WONDER website. Beginning with the 1999 data year, the National Center for Health Statistics adopted the age distribution of the year 2000 population of the United

States as the standard population for the purpose of age adjustments. The age‐adjusted mortality rates are provided by the CDC WONDER website and are calculated yearly using the direct standardization method based on the age group weights from the 2000 US population. Detailed data on the calculation of the age‐adjusted mortality rates can be found online (https://wonder.cdc.gov/wonder/help/mcd‐expanded.html#Age‐Adjusted%20Rates). We examined temporal trends in obesity‐related cardiovascular mortality by race, reporting both crude and age‐adjusted mortality rates as well as percentage change between 1999 to 2020. For comparison, we calculated trends in all cardiovascular mortality (regardless of relation to obesity) over the same period. Analysis was performed using IBM SPSS version 26.

## Results

### Population Characteristics

**Figure 1:**
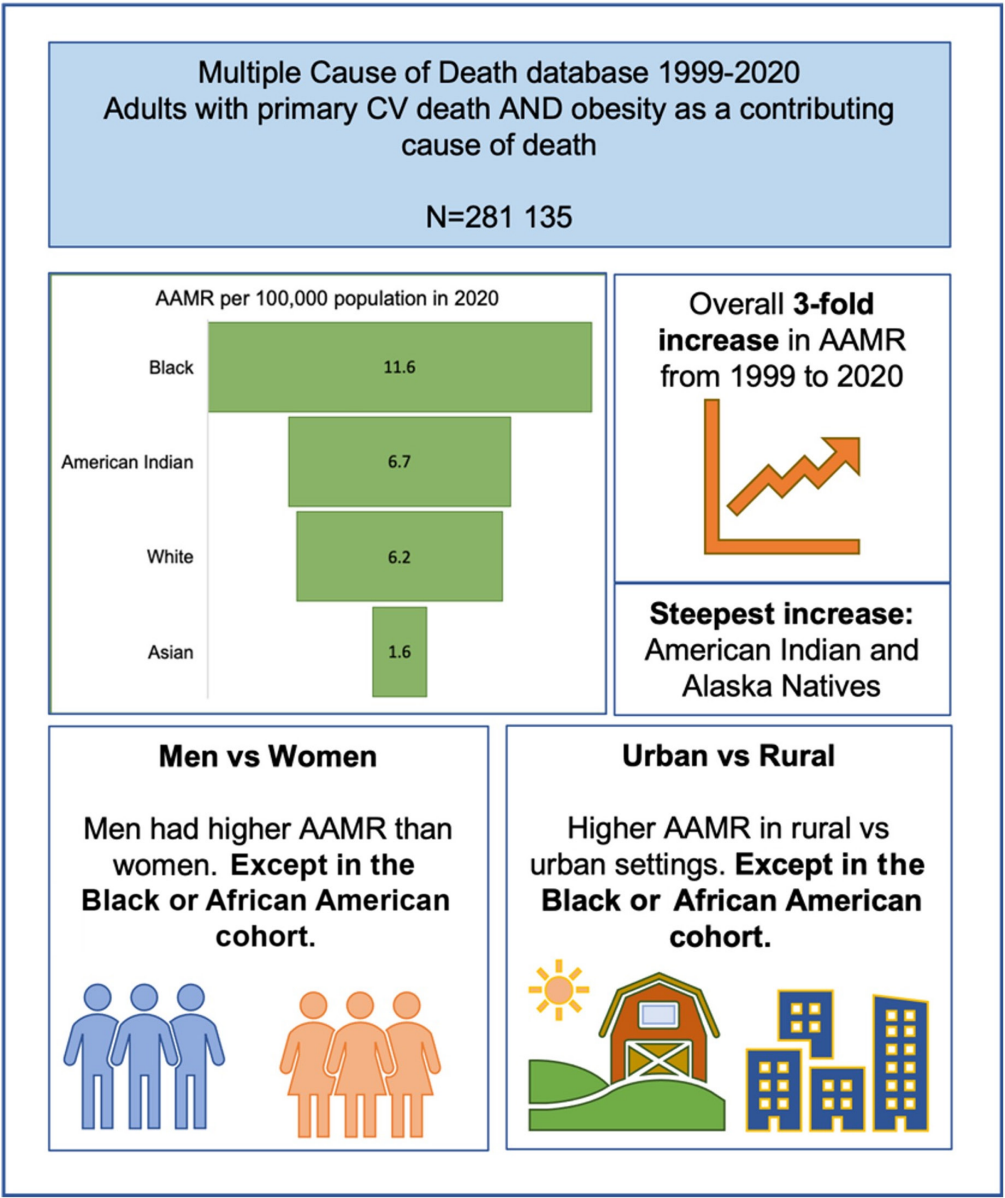
Overview of study findings. Asian refers to Asian or Pacific Islander. American Indian refers to American Indian or Alaska Native. AAMR indicates age‐adjusted mortality rate; and CV, cardiovascular.

Over the entire study period (1999 to 2020), we identified 281 135 primary CVD deaths with obesity recorded as a contributing cause of death (Table 1, Figure 1). Among these, 122 697 (43.6%) deaths were in women. The sample included 78.1% (n=219 561) White, 19.8% (n=55 623) Black, 1.1% (n=3223) Asian or Pacific Islander, and 1.0% (n=2728) American Indian or Alaska Native individuals.

**Table 1:** Population Characteristic and Obesity‐Related Cardiovascular Mortality in the United States Between 1999 and 2020, Stratified by Race

Characteristic	American Indian or Alaska Native			Asian or Pacific Islander			Black			White		
	No. of deaths	Crude rate*	Age‐adjusted rate*	No. of deaths	Crude rate*	Age‐adjusted rate*	No. of deaths	Crude rate*	Age‐adjusted rate*	No. of deaths	Crude rate*	Age‐adjusted rate*
Overall	2728	3.1	3.8	3223	0.9	0.9	55 623	6.1	6.7	219 561	4.1	3.6
Primary cause of death												
Ischemic heart diseases	1281	1.4	1.8	1546	0.4	0.4	21 541	2.3	2.6	115 534	2.2	1.8
Hypertensive diseases	648	0.7	0.9	716	0.2	0.2	17 642	1.9	2.1	42 146	0.8	0.7
Heart failure/cardiomyopathy	260	0.3	0.4	345	0.1	0.1	4931	0.5	0.6	17 386	0.3	0.3
Cerebrovascular diseases	91	0.1	0.1	156	0.1	0.1	1870	0.2	0.2	7287	0.1	0.1
Sex												
Men	1626	3,7	4,6	2086	1.2	1.2	25 380	5.8	6.6	129 346	4.9	4.4
Women	1102	2.5	3.0	1137	0.6	0.6	30 243	6.3	6.7	90 215	3.3	2.8
Age, y†												
15–24	39	0.3	N/A	57	0.1	N/A	861	0.6	N/A	1185	0.2	N/A
25–34	182	1.4	N/A	277	0.4	N/A	3932	2.9	N/A	6695	0.9	N/A
35–44	507	4.2	N/A	607	1.0	N/A	9260	7.3	N/A	20 513	2.8	N/A
45–54	678	6.4	N/A	808	1.6	N/A	14 527	12.	N/A	46 059	6.1	N/A
55–64	717	9.6	N/A	719	2.0	N/A	14 021	16.3	N/A	62 709	9.8	N/A
65–74	420	10.8	N/A	466	2.2	N/A	8424	17.0	N/A	48 743	11.2	N/A
75–84	143	8.7	N/A	221	2.1	N/A	3509	14.2	N/A	25 586	9.8	N/A
≥85	39	7.5	N/A	68	1.9	N/A	1039	11.7	N/A	7981	7.5	N/A
US region												
Northeast	98	1.2	1.4	311	0.4	0.5	9915	6.1	6.5	36 942	3.8	3.2
Midwest	420	3.2	4.1	172	0.4	0.5	10 296	6.3	7.2	53 723	4.3	3.8
South	922	3.5	4.1	364	0.5	0.5	28 905	5.8	6.3	72 400	3.8	3.3
West	1288	3.1	4.0	2366	1.3	1.3	6507	7.2	8.3	56 496	4.5	4.2
Urbanization												
Urban	1579	2.5	3.2	2980	0.8	0.9	50 787	6.1	6.8	175 170	3.9	3.5
Rural	1149	4.5	5.4	243	2.0	2.2	4836	5.5	5.9	44 391	5.0	4.1

* Death rates are per 100 000 population.

† Because age adjustment is based on 10‐year age groups, age‐adjusted data on specific age groups are not available.

NA, not available.

### Temporal Trends in Cardiovascular Mortality

In the whole cohort, there was a reduction in the crude rate of all cardiovascular deaths between 1999 and 2020 (−17.6%), consistent across all races (Table 2). The degree of reduction was greater when calculating age‐adjusted rates (−36.0%). The largest decrease in age‐adjusted cardiovascular mortality was among American Indian or Alaska Native individuals (−42.9%), and the smallest reduction was observed among Black individuals (−31.9%).

**Table 2:** Temporal Trends in Overall and Obesity‐Related Cardiovascular Mortality in the United States Between 1999 and 2020, Stratified by Race

Racial stratification	No. of deaths			Crude death rate*			Age‐adjusted rate*		
	1999	2020	% Change	1999	2020	% Change	1999	2020	% Change
Overall cardiovascular mortality	954 339	928 741	−2.7%	342	281.9	−17.6%	350.8	224.4	−36.0%
American Indian or Alaska Native	3134	5518	+76.1%	110.6	112.6	+1.8%	263.7	150.7	−42.9%
Asian or Pacific Islander	13 087	28 896	+120.8%	115.3	128.9	+11.8%	225	134.6	−40.2%
Black	105 309	130 008	+23.5%	291.1	275.6	−5.3%	450	306.6	−31.9%
White	832 809	764 319	−8.2%	364.2	299.7	−17.7%	343.3	219.1	−36.2%
Obesity‐related cardiovascular mortality	6141	25 794	+320.0%	2.2	7.8	+254.5%	2.2	6.6	+200.0%
American Indian or Alaska Native	25	296	+1084.0%	0.9	6	+566.7%	1.3	6.7	+415.4%
Asian or Pacific Islander	36	381	+958.3%	0.3	1.7	+466.7%	0.4	1.6	+300.0%
Black	1190	5398	+353.6%	3.3	11.4	+245.5%	4.2	11.6	+176.2%
White	4890	19 719	+303.3%	2.1	7.7	+266.7%	2.1	6.2	+195.2%

* Death rates are per 100 000 population.

**Figure 2:**
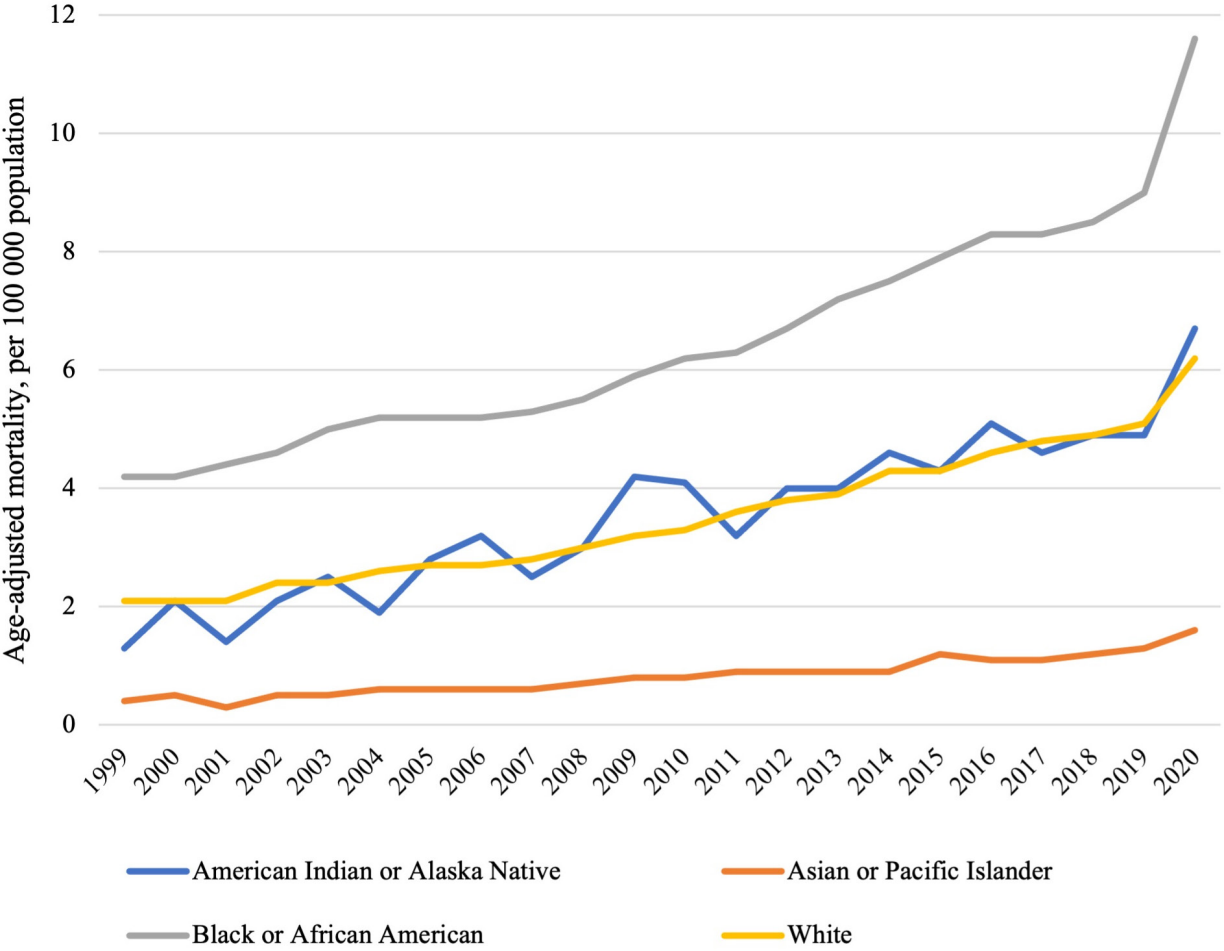
Temporal trends in obesity‐related age‐adjusted cardiovascular mortality in the United States between 1999 and 2020, stratified by race.

In contrast to overall cardiovascular mortality trends, age‐adjusted obesity‐related cardiovascular mortality tripled from 2.2 to 6.6 per 100 000 population from 1999 to 2020 (+200.0%). This increasing trend was consistent across all racial groups (Table 2, Figure 2). Throughout the entire study period, the age‐adjusted rate of obesity‐related cardiovascular mortality was greater in Black individuals than in any other racial group (Figure 2). The highest obesity‐related cardiovascular mortality was observed in Black individuals (age‐adjusted mortality of 4.2–11.6 per 100 000 population in 1999 to 2020), whereas the greatest increase in mortality was observed among American Indian or Alaska Native individuals (1.3–6.7 per 100 000 population, +415%). The rate of increase in age‐adjusted mortality appeared to increase in the latter years of the study (Figure 2). The lowest age‐adjusted obesity‐related cardiovascular mortality was observed among Asian or Pacific Islander individuals (0.4–1.6 per 100 000 population in 1999 to 2020).

### Cardiovascular Causes of Death

**Figure 3:**
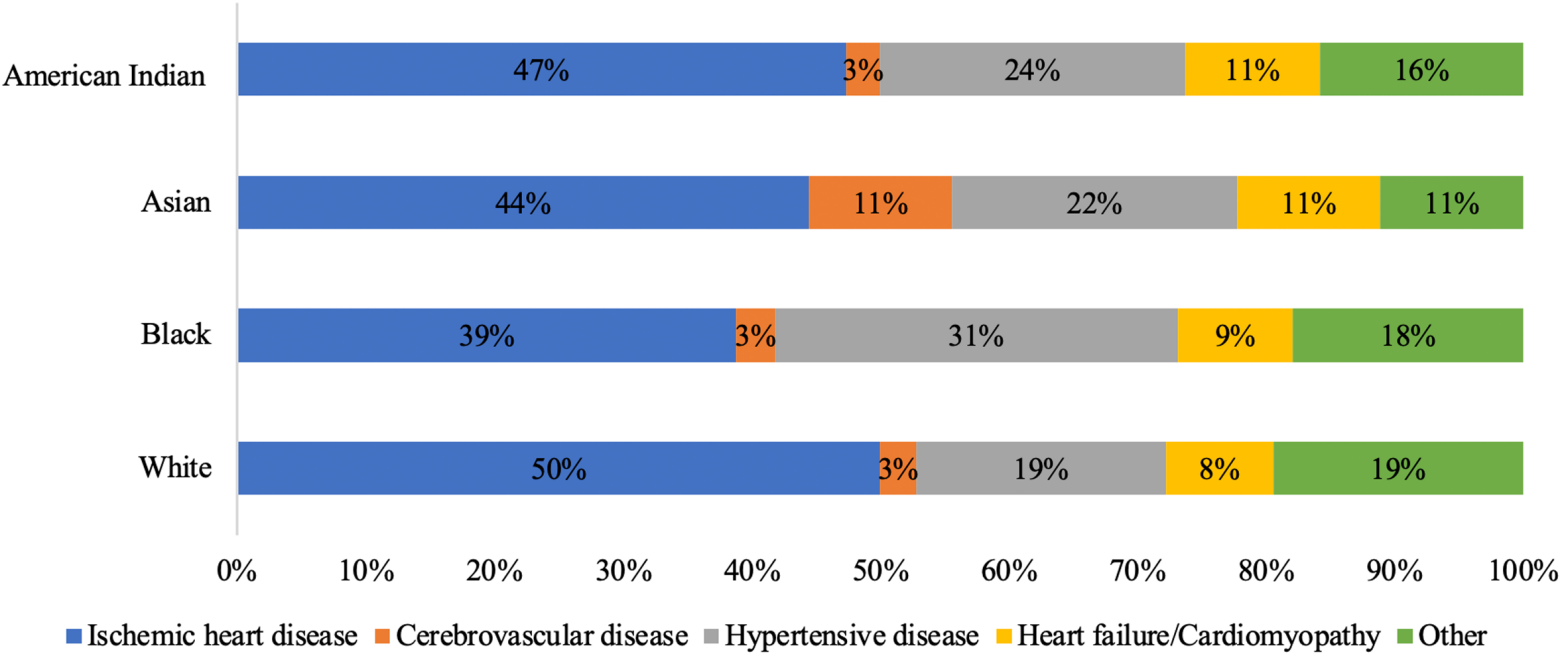
Distribution of primary cardiovascular causes of death in individuals with obesity as a contributory cause, stratified by racial group. American Indian refers to American Indian or Alaska Native; Asian refers to Asian or Pacific Islander; Black refers to Black or African American.

Ischemic heart disease was the most common cardiovascular cause of death across all races (Figure 3); it was most common among individuals from White (50%) and American Indian or Alaska Native (47%) racial groups. The second most common cause of death was hypertensive disease, which occurred most frequently among Black (31%) and American Indian or Alaska Native (24%) individuals and least commonly among White (19%) individuals. Cerebrovascular disease occurred most commonly as a primary cause of death among individuals of Asian or Pacific Island race, contributing 11% of obesity‐related cardiovascular deaths in this cohort, compared with 3% in all other racial groups (Figure 3).

### Cardiovascular Mortality by Age and Sex

As expected, in the whole sample, there was a greater proportion of men (n=158 438, 56.4%) than women (n=122 697, 43.6%). This differential sex representation was most pronounced among Asian or Pacific Islander and White individuals, with women comprising only 35.3% (n=1137) and 41.1% (n=90 215) of these cohorts, respectively (Table 1). In contrast to all other racial groups, there was a greater proportion of women (n=30 243, 54.4%) than men among Black individuals.

Age‐adjusted obesity‐related cardiovascular mortality was higher among Black individuals (6.7 per 100 000 population; Table 1) than any other racial group, followed by American Indian or Alaska Native (3.8 per 100 000 population) individuals, and lowest among Asian or Pacific Island individuals (0.9 per 100 000 population).

**Figure 4:**
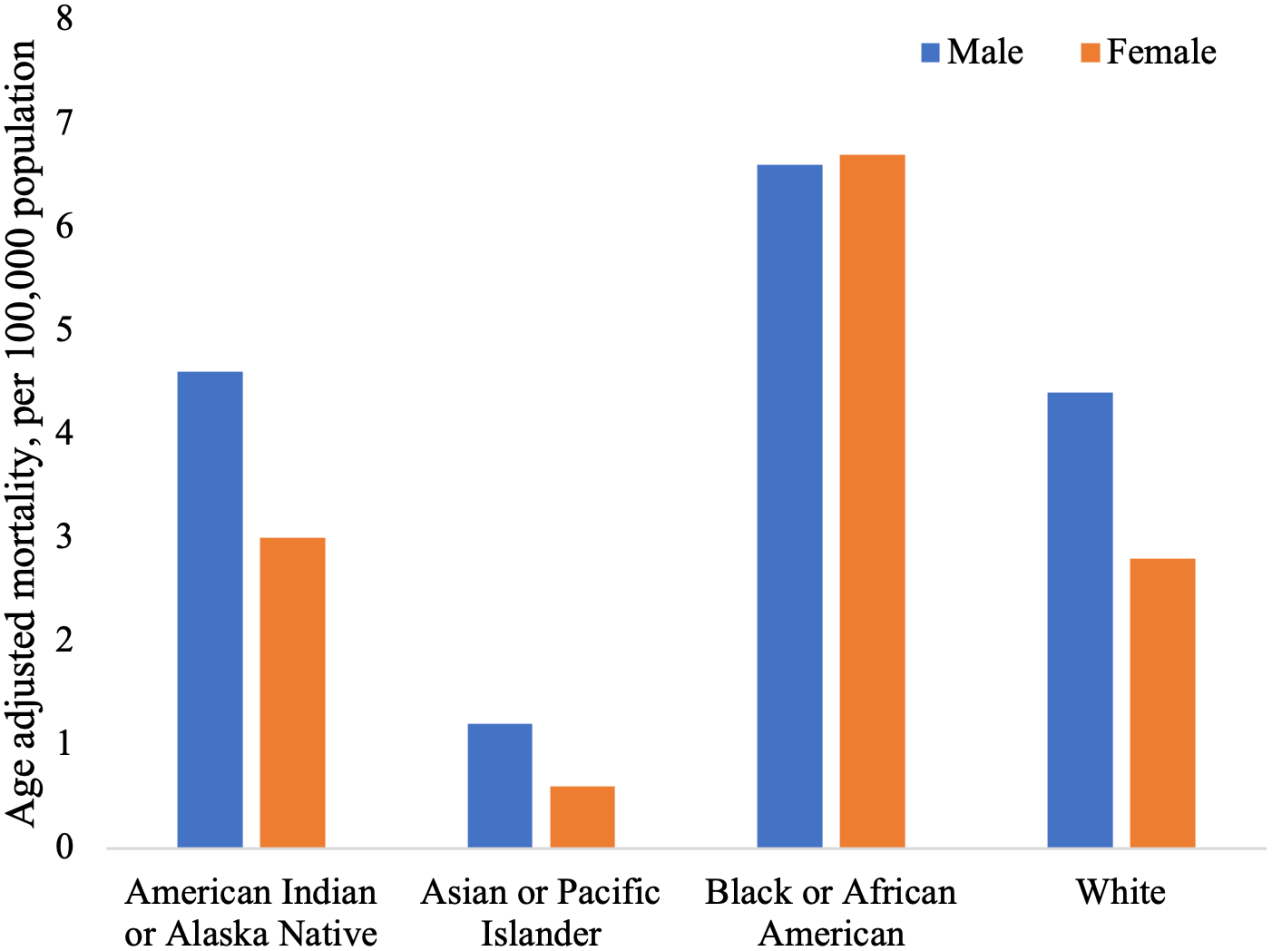
Age‐adjusted obesity‐related cardiovascular mortality rate, stratified by sex and race.

Among Black individuals, the age‐adjusted mortality rate was slightly higher among women than men (6.7 versus 6.6 per 100 000 population; Table 1, Figure 4), whereas across all other races, age‐adjusted mortality rates were higher in men (range, 4.6–1.2 per 100 000 population) compared with women (range, 2.8–0.6 per 100 000 population).

Across all racial groups, the highest crude mortality rates were observed in individuals 65 to 74 years old (Table 1, Figure S1), and the lowest rates were in those <55 years old.

### Cardiovascular Mortality by Place of Residence

**Figure 5:**
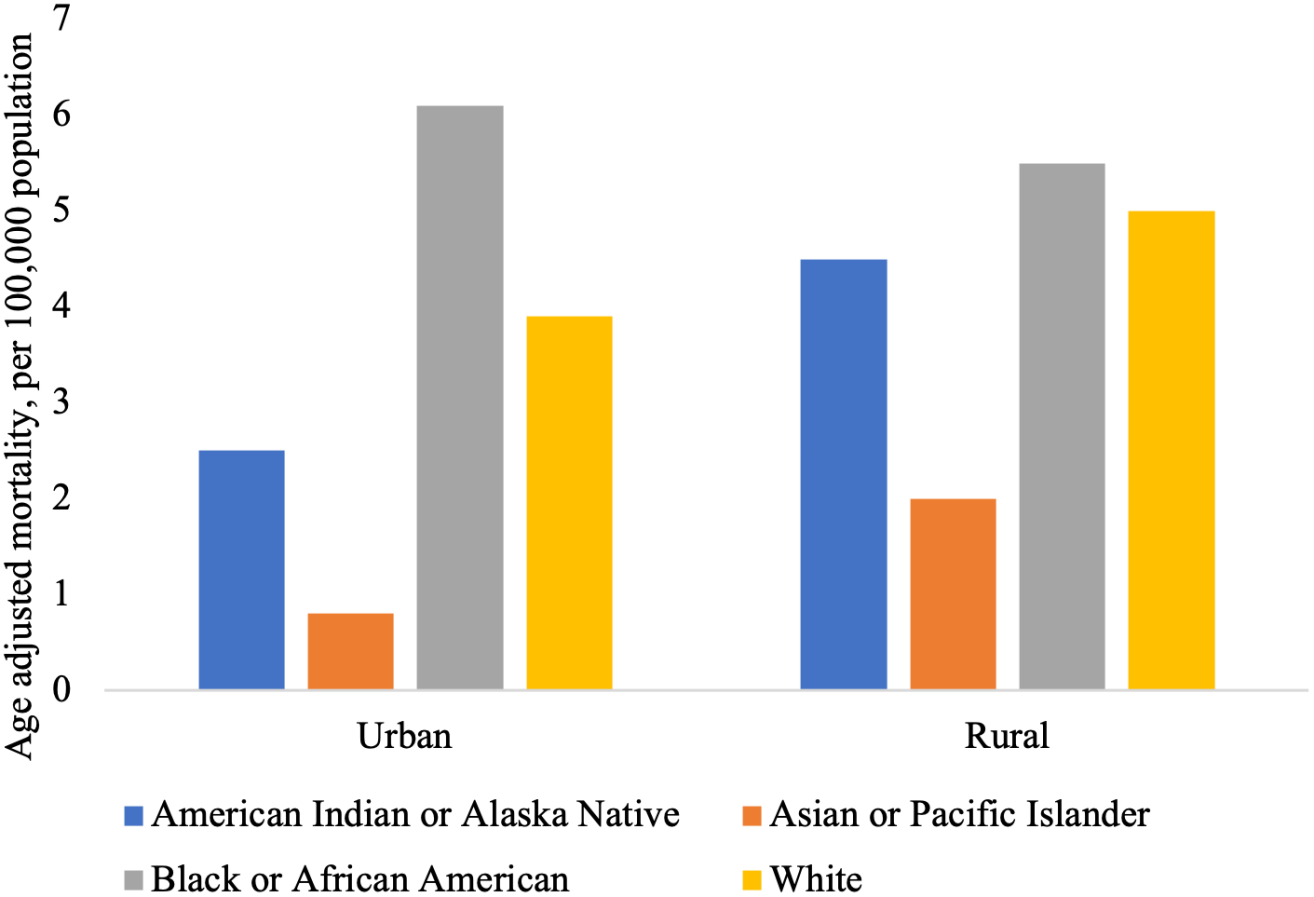
Age‐adjusted obesity‐related cardiovascular mortality rate, stratified by urbanization and race.

Black individuals living in urban settings experienced higher rates of age‐adjusted obesity‐related cardiovascular mortality than those living in rural regions (6.8 versus 5.9 per 100 000 population). The reverse pattern was observed across all other racial groups, with greater age‐adjusted death in rural (range, 2.2–5.4 per 100 000 population) than in urban (range, 0.9–3.5 per 100 000 population) settings (Table 1, Figure 5).

Among Black, White, and Asian or Pacific Islander individuals, the highest age‐adjusted mortality rate was observed in those living in the West region, with age‐adjusted mortality rates of 7.2, 4.5, and 1.3 per 100 000 population, respectively (Table 1). Among American Indian or Alaska Native individuals, higher mortality was observed in the South and Midwest regions (4.1 per 100 000 population in both).

## Discussion

Obesity is a complex global public health problem. Although the increasing prevalence of obesity has been highlighted in many countries, few researchers have examined the population burden of cardiovascular mortality related to obesity, and none have considered differential trends by race.

We observed significant increasing trends of cardiovascular deaths related to obesity across racial groups, with an overall 3‐fold increase in age‐adjusted mortality between 1999 and 2020. This observation reflects the health consequences of the rising burden of obesity in the US population and is consistent with previous work highlighting CVD as the most common cause of obesity‐related death.^13^ This trend was in contrast to general cardiovascular mortality, which consistent with existing reports,^10^ declined significantly over the 2 decades studied.

In our analysis, individuals of Black background had higher age‐adjusted obesity‐related cardiovascular mortality rates than any other racial group throughout the entire study period. This observation likely reflects the greater population burden of obesity among Black individuals, who, according to the latest estimates, have the highest estimated prevalence of obesity of any racial group in the United States.^14^ A further possibility is that there is a disproportionate number of Black individuals with severe obesity phenotypes, which are associated with a greater risk of cardiovascular mortality.^15^ Furthermore, some researchers have suggested that the adverse impact of obesity, at any given body mass index, is higher in some racial groups than others. For instance, in a prospective analysis of 78 419 individuals from the Nurse's Healthy Study, Shai et al^16^ report higher relative risk of diabetes in Black patients compared with White patients for the same increase in body mass index. However, these observations have not been replicated in different settings. For example, Boggs et al found similar patterns in associations between obesity and death in White and Black people.^17^ A further key consideration is that the adverse health consequences of obesity are augmented in Black individuals by other coexisting societal sources of disadvantage, and that it is the constellation of these different sources of disadvantage that altogether drives higher cardiovascular mortality rates related to obesity. Few studies make a concerted effort to characterize important social factors such as racism and health inequalities in these relationships. Such social determinants of health are often incompletely captured, and this makes disentangling their effect from that of obesity extremely challenging. There is a need for greater focus on the health care needs of individuals from underrepresented populations to understand the different social factors that drive poorer health in these communities and to design strategies that may be implemented to address these issues at social, political, and health care levels. These factors have particular relevance in the context of obesity, given the complex network of social and biological factors that drive this condition. As we demonstrate in our study, the increase in obesity translates to greater burden of related diseases, in our case cardiovascular mortality. Thus, understanding and addressing the root causes of obesity and its disparities across different groups is a growing public health priority.

Another notable observation from our analysis resulted from the comparison of sex differences within racial groups. As expected from previous epidemiologic reports,^15^, ^18^ in the whole sample, men had a greater proportion of obesity‐related cardiovascular mortality cases and a higher age‐adjusted mortality rate. This was consistent across all racial groups, except in Black individuals, in whom women had a greater proportion of obesity‐related cardiovascular deaths and higher age‐adjusted mortality rate than the men. This is an unexpected finding, because in unselected epidemiologic reports, women generally have lower cardiovascular risk compared with men.^18^ The disproportionate adverse health experiences of Black women have been highlighted across several settings including mental health, maternal outcomes, and cardiovascular health.^19^ Black women are among the most vulnerable cohorts in society, facing disadvantage across a host of social and economic measures, which has been shown to translate into significant health inequalities.^20^ A growing body of work highlights the adverse chronic psychosocial stressors faced by Black women, exacerbated by systematic racism and misogyny.^21^ It is important to note that the significant inequalities in health outcomes experienced by Black women cut across social class and socioeconomic status.^19^ Notably, middle‐class educated Black women who endorse an obligation to show resilience and suppress emotion in the face of racial microaggressions have been shown to experience increased psychosocial distress,^22^ which in turn has been linked to higher cardiovascular risk in this group.^23^ Furthermore, relevant to our study, Black women with greater obesity were assessed to have greater psychological and sociological barriers to weight management.^24^ Previous work has demonstrated that a greater burden of obesity in Black women (compared with Black men) exists even in early childhood and adolescence. In an analysis of obesity rates among children 2 to 19 years old, Ogden et al report higher obesity rates among Black girls than among Black boys.^25^ The greatest difference was among adolescents (12–19 years old), with the rate of 28.2% among Black girls and 22.0% among Black boys.^25^ These findings highlight the importance of early life factors in determining health outcomes in adulthood, and in particular the tracking of childhood obesity trends into older ages. The observations may also reflect, in part, cultural factors among Black communities that result in girls and women having higher rates of obesity than boys and men.

Our observations of greater obesity‐related cardiovascular death among Black women compared with men reflects the cultural and sociopolitical context outlined. Our results indicate that there are disparities in the prevalence and severity of obesity among Black women (with women having greater obesity), which do not exist (at least to the same extent) within other racial groups, and that these disparities translate into greater cardiovascular mortality related to obesity. Health care strategies aimed at addressing obesity must tackle as a priority the social determinants that drive health inequalities in Black women.

In our analysis, ischemic heart disease was the most common obesity‐related cardiovascular cause of death across all racial groups. This is in keeping with widely established mechanistic links between obesity and key atherosclerotic risk factors, such as diabetes, hypertension, and dyslipidemia.^8^ Furthermore, growing research indicates that obesity itself is an independent driver of poorer cardiovascular health and has a likely causal role in driving a range of CVDs.^26^ Our observations also partly reflect the high contribution of ischemic heart disease to CVD in general, regardless of obesity context.^27^

The second most common cause of obesity‐related cardiovascular mortality in our study was hypertensive disease. The dominance of hypertension as a cause of death in our study is in keeping with previous studies that highlight obesity as a mechanistic driver of hypertension.^26^, ^28^, ^29^ We further observed that hypertensive deaths occurred substantially more frequently in Black individuals than in any other racial group. Previous work has highlighted the higher prevalence, younger age of onset, poorer control, and more severe adverse outcomes related to hypertension in Black populations.^30^, ^31^, ^32^ A further contributing factor is underrepresentation of Black individuals in hypertension trials, likely driven by socioeconomic barriers and absence of dedicated outreach. Furthermore, existing evidence indicates unequal practices in clinical decision making for racial and ethnic groups, which are influenced by patient‐ and system‐level issues, bias and racism, patient values, and communication.^33^ There is need for further research to establish equitable and evidence‐based clinical decision making and to optimize hypertension control among diverse racial groups. Our findings add to the existing literature in highlighting the role of obesity in driving hypertension‐related mortality and its disproportionate impact on Black populations.

Our findings additionally demonstrate the increasing burden of cardiovascular mortality related to obesity in American Indian or Alaska Native individuals, with this cohort showing the greatest temporal increase in age‐adjusted mortality of any racial group studied. These populations are historically underserved, and our results highlight the impact of systematic social disadvantage as manifested through obesity and cardiovascular death. There is need for dedicated action to understand the health and social care needs of American Indian or Alaska Native individuals and to guide the development of public health strategies that may best serve these communities. A quarter of American Indian individuals live below the federal poverty line.^34^ As such, the social determinants of cardiovascular health are of high importance in these communities, driven by inequalities in social structures.^34^ A core component of any public health strategy to reduce obesity should include addressing social determinants of health at a community level.^34^, ^35^

In our study, individuals of Asian or Pacific Islander race had lower rates of age‐adjusted obesity‐related cardiovascular mortality than any other racial group. Although this remained the case throughout the 2 decades studied, this cohort also had the second highest temporal increase in age‐adjusted mortality, indicating that obesity is increasingly impacting health outcomes even in this relatively low‐risk racial group. It is worth noting that this racial group is highly heterogenous, and our findings may not apply to all individuals classified within this composite racial group. More granular analysis of individual racial groups is needed for definitive assessment of trends and risk in this cohort.

We also observed disparities in obesity‐related cardiovascular mortality between individuals living in urban versus rural settings. In Black individuals, we observed greater age‐adjusted mortality rates in urban compared with rural settings, although in all other racial groups the reverse was observed. Previous work has consistently shown that health outcomes in general are poorer in rural settings, largely due to reduced accessibility of health care.^36^ Our study is the first to report racial differences in this relationship. A potential explanation for our observations may be higher levels of socioeconomic disadvantage experienced by Black communities in urban areas compared with rural settings, which is driving widening of racial inequalities across several domains including health care.

## Strengths and Limitations

The CDC WONDER resource permitted access to population‐level mortality records for the entire US population over the time period of study, ensuring high completeness and representativeness of our analysis sample. The database is limited to US residents, which means that deaths in nonresident aliens or deaths occurring outside the United States are not captured. It is expected that such numbers are small and would not substantially impact the results observed. The cause of death was recorded using *ICD‐10* codes, which permitted standardized ascertainment of our outcome of interest across the entire data set. However, such records may also be subject to miscoding or misdiagnosis errors. Another important limitation is that we were not able to adjust for baseline comorbidities, nor for other important potential confounders, such as socioeconomic status, insurance type, education, and occupation. Furthermore, the data set does not permit analytic analyses to investigate the underlying drivers of the trends and distributions observed. Clearly, these would be important subjects for further study.

## Conclusions

In this large population‐level analysis of mortality data, we demonstrate a substantial rise in cardiovascular mortality related to obesity in the US population over the past 2 decades between 1999 and 2020. This is in contrast to general cardiovascular mortality trends, which have steadily declined over the same time period. Our results highlight Black individuals, in particular women from these communities, as being disproportionately impacted. We further identify American Indian or Alaska Native individuals as a cohort in whom the risk of obesity‐related cardiovascular mortality is rising most rapidly. There is need for dedicated health strategies aimed at individual communities to better understand and tackle the social determinants of obesity and to design interventions that may alleviate the population burden of both obesity and cardiovascular disease.

## Sources of Funding

Z.R.‐E. recognizes the National Institute for Health Research Integrated Academic Training program, which supports her Academic Clinical Lectureship post; she was also supported by a British Heart Foundation Clinical Research Training Fellowship (FS/17/81/33318). K.B. receives research support from the National Heart, Lung, and Blood Institute (K01HL142848, R56HL159216, and L30HL14888).

## Disclosures

None.

## Supporting information

Figure S1
