# Supplementary material for: Racial Disparities in Obesity‐Related Cardiovascular Mortality in the United States: Temporal Trends From 1999 to 2020
Source: J Am Heart Assoc. 2023 Sep 6;12(18):e028409. doi: 10.1161/JAHA.122.028409 (PMC10547286; doi:10.1161/JAHA.122.028409)

SUPPLEMENTAL MATERIAL

Figure S1. Crude rate of obesity-related cardiovascular mortality, stratified by age and ethnicity.

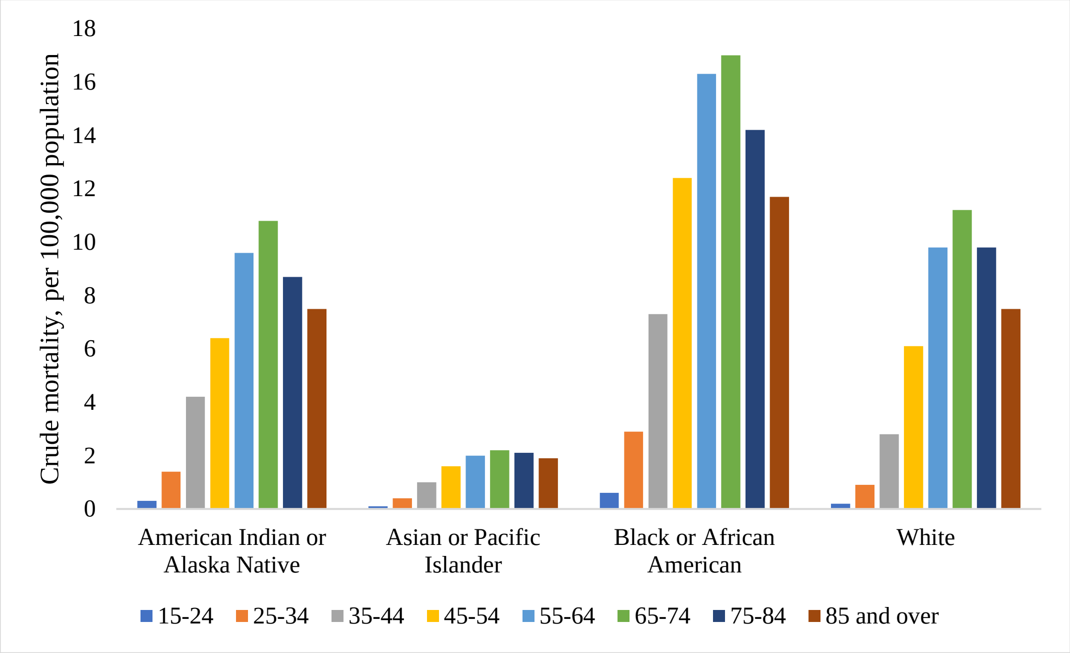

Supplement: Supplementary file 1 — Figure S1 [file JAH3-12-e028409-s001.pdf]
